# Ginsenoside-Rb1 Protects Hypoxic- and Ischemic-Damaged Cardiomyocytes by Regulating Expression of miRNAs

**DOI:** 10.1155/2015/171306

**Published:** 2015-05-17

**Authors:** Xu Yan, Jinrong Xue, Hongjin Wu, Shengqi Wang, Yuna Liu, Sidao Zheng, Chengying Zhang, Cui Yang

**Affiliations:** ^1^Beijing Haidian Hospital, Haidian Section of Peking University Third Hospital, 29 Zhongguancun Dajie, Haidian District, Beijing 100080, China; ^2^Postdoctoral Workstation of the Zhongguancun Haidian Science Park, No. 6 Sijiqing Road, Haidian District, Beijing 100195, China; ^3^Beijing Hospital of Integrated Traditional Chinese and Western Medicine, Dongjie 3, Yongding Road, Haidian District, Beijing 100039, China; ^4^Department of Cardiovascular Surgery, Beijing Aortic Disease Center, Beijing Anzhen Hospital, Capital Medical University, No. 2 Anzhen Road, Chaoyang District, Beijing 100029, China; ^5^Beijing Institute of Radiation Medicine, Beijing 100850, China

## Abstract

Ginsenoside (GS-Rb1) is one of the most important active compounds of ginseng, with extensive evidence of its cardioprotective properties. However, the miRNA mediated mechanism of GS-Rb1 on cardiomyocytes remains unclear. Here, the roles of miRNAs in cardioprotective activity of GS-Rb1 were investigated in hypoxic- and ischemic-damaged cardiomyocytes. Neonatal rat cardiomyocytes (NRCMs) were first isolated, cultured, and then incubated with or without GS-Rb1 (2.5–40 *μ*M) *in vitro* under conditions of hypoxia and ischemia. Cell growth, proliferation, and apoptosis were detected by MTT and flow cytometry. Expressions of various microRNAs were analyzed by real-time PCR. Compared with that of the control group, GS-Rb1 significantly decreased cell death in a dose-dependent manner and expressions of mir-1, mir-29a, and mir-208 obviously increased in the experimental model groups. In contrast, expressions of mir-21 and mir-320 were significantly downregulated and GS-Rb1 could reverse the differences in a certain extent. The miRNAs might be involved in the protective effect of GS-Rb1 on the hypoxia/ischemia injuries in cardiomyocytes. The effect might be based on the upregulation of mir-1, mir-29a, and mir-208 and downregulation of mir-21 and mir-320. This might provide us a new target to explore the novel strategy for ischemic cardioprotection.

## 1. Introduction

MicroRNAs (miRNAs) are a kind of conservative single stranded noncoding RNA molecules found in animals, plants, virus, and other organisms and regulate proliferation, differentiation, apoptosis and metabolism, and other cellular processes [1, 2]. In recent years, investigations found that miRNAs play an important role in the development of cardiovascular diseases and may become a possible new target in the treatment of cardiovascular diseases [3].

Myocardial ischemic injury resulting from severe impairment of the coronary blood supply is a severe stress that leads to the loss of cardiomyocytes by apoptosis and necrosis. MicroRNAs have been proved to be potential biomarkers for ischemic heart disease, such as mir-1, mir-133, mir-208, and mir-499 [4–6]. Several studies also demonstrated that miRNAs dysregulation has a key role in the ischemic heart disease process. Various miRNAs can regulate gene expression at the posttranscriptional level by either translational repression of a target mRNA or degradation of myocardial death induced mir expression in the genome of most eukaryotes. Many studies have revealed that microRNAs could be the therapeutic targets for common used drugs or new drug design, which could be potentially used as complementary and alternative interventions for the treatment of ischemic heart disease.

Ginseng, the root of* Panax ginseng* C.A. Meyer, has been widely used in traditional Chinese medicine for several thousand years [7]. Ginseng plays critical roles in the endocrine, immune, central nervous, and especially the cardiovascular systems [8]. Ginsenoside-Rb1 (GS-Rb1), a major pharmacological extract, is one of the most important active compounds of ginseng, with extensive evidence of its cardioprotective properties. Numerous studies have indicated that the cardioprotective effect of GS-Rb1 was affected by multiple pathways [9]. However, regulatory roles of GS-Rb1 in miRNA during cardiomyocytes apoptosis are rarely studied. In the present investigation, rat myocardial cells were first isolated, cultured, and incubated with GS-Rb1* in vitro* under conditions of hypoxia and ischemia. The protective roles of GS-Rb1 were explored, and five circulation-related microRNAs' expression change in each group was then analyzed by poly(A) tailing SYBR Green real-time PCR.

## 2. Materials and Methods

### 2.1. Materials

GS-Rb1 (catalog number 110704) purchased from National Institutes for Food and Drug Control was dissolved in phosphate-buffered saline (PBS) to create a stock solution for subsequent dilution. miRNA extracting kit (CW0627), reverse transcription kit (CW2141), and fluorescent quantitation PCR (CW2142) kit were all purchased from BeiJing Cowin Biotech Co., Ltd. Annexin V-FITC/PI kit was purchased from Kaiji Biological Engineering Institute.

### 2.2. Isolation and Culture of Neonatal Rat Cardiomyocytes

All experiments were approved by the Beijing Ethics Committee for the Use of Experimental Animals. Primary cultures of NRCMs from 12–24-hour-old Sprague Dawley rats (Vital River Laboratories, Beijing, China) were prepared by means of gentle serial trypsinization as described before with slight modification [10]. Briefly, the ventricular myocardium was removed and cut into small pieces (1-2 mm^3^). The ventricles obtained were washed three times in cold PBS and digested 5 times for 5 min each at 37°C with 0.18% (w/v) trypsin and 0.01% EDTA. Addition of an equal volume of cold Dulbecco's modified Eagle's medium (DMEM) containing 10% (v/v) newborn calf serum was used to terminate the digestion. Then, cells were collected by centrifugation for 10 min at 1000 g/min at room temperature. Cells were then resuspended in DMEM with 20% (v/v) FCS for 60 min to facilitate separation of ventricular myocytes from the faster-attaching nonmyocytes. The NRCMs were then collected and plated in collagen-coated 96- or 6-well plates and maintained at 37°C in a 5% CO_2_/95% air humidified incubator in DMEM containing 10% (v/v) fetal bovine serum, 100 U/mL penicillin, and 100 mg/mL streptomycin. The following experiments used spontaneously beating cardiomyocytes 48–72 h after plating.

### 2.3. Hypoxia/Ischemia Treatment

To generate hypoxic/ischemic conditions, culture medium was replaced with DMEM (no glucose) (Gibco, Grand Island, USA) with serum free. NRCMs with or without GS-Rb1 were then placed in a W-Zip package (an0010, Oxide Anaerobe Pouch System), which was capable of depleting the concentration of O_2_ down to 10% in 2 h. The sealed package was subsequently placed into a 37°C incubator for 12 h after pretreatment with GS-Rb1 for 6 h. The control plates were kept in normoxic conditions for the corresponding times.

### 2.4. MTT Assay

NRCMs viability was determined using the MTT assay. Cardiomyocytes were plated on 96-well dishes at 2 × 104 cells/well. MTT at 5 mg/mL was added to each well immediately after 12 h of hypoxia/ischemia. Plates were incubated for 4 h at 37°C. The medium was aspirated from each well and 100 *μ*L of DMSO was added to dissolve the formazan crystals. The optical density of each well was read at 492 nm using a Microplate Reader (Bio-Rad, Hercules, CA). Results are given as percentages of the control group taken as 100%.

### 2.5. Apoptosis Assay by Annexin V/PI Staining

NRCMs with different concentration of GS-Rb1 exposed to hypoxia/ischemia conditions were harvested and washed with PBS. The percentage of normal nonapoptotic cells was measured by double supravital staining with Annexin V and PI, using an Annexin V Apoptosis Detection kit (KeyGen, Nanjing, China). Flow cytometric analysis used a Cytomics FC500 flow cytometer with CXP software (Beckman Coulter, Fullerton, USA), the operator being blind to the group assignment.

### 2.6. Detection of miRNA Expression Using Poly(A) Tailing SYBR Green Real-Time PCR

Total RNA (5 *μ*g) was treated with DNase I for 15 minutes at 22°C (Invitrogen) and then poly(A)-tailed using poly(A) polymerase (NEB) at 37°C for 1 hour. The final reaction mixture was extracted with phenol/chloroform, precipitated with isopropanol, and redissolved in 25 *μ*L of diethylpyrocarbonate-treated water. Poly(A)-tailed RNA (6 *μ*L) was reverse-transcribed into first-strand cDNA using a miRNA cDNA kit (cw2141, BeiJing Cowin Biotech Co., Ltd.). For PCR, 30 ng of cDNA was used as a template in each reaction using miRNA Real-Time PCR Assay Kit (cw2142, BeiJing Cowin Biotech Co., Ltd.). The forward primer for each miRNA was

 mir-1: 5′-GCGTGGAATGTAAAGAAGTGTGTATAAA-3′;
 mir-29a: 5′-TAGCACCATCTGAAATCGGTTAAAA-3′;
 mir-208: 5′-ATAAGACGAGCAAAAAGCAAAAAAAA-3′;
 mir-21: 5′-GCTAGCTTATCAGACTGATGTTGAAAA-3′;
 mir-320: 5′-AAGCTGGGTTGAGAGGGCGA-3′; U6 small noncoding RNA sequence was amplified as an internal control using the primers 5′-CTCGCTTCGGCAGCACA-3′ (forward) and 5′-AACGCTTCACGAATTTGCGT-3′ (reverse).

The anneal temperature was 63°C. The SYBR Green-based real-time PCR was performed using ABI 7500 Real-Time PCR System (Applied Biosystems). The relative expression of miRNA was calculated based on the formula: 2^−(ΔCtmiR−ΔCtU6)^.

### 2.7. Statistical Analysis

The data obtained were presented as the mean ± SEM of 3 independent experiments. The relationship between two factors was analyzed by Pearson correlation analysis. Bootstrap was used in paired samples tests. Group results were analysed for variance using ANOVA. Two groups were compared by Student's *t*-test. All analyses used GraphPad Prism 5.0 software. A *P* < 0.05 indicated that the difference was statistically significant. A *P* < 0.01 indicated that the difference was extremely and statistically significant.

## 3. Results

### 3.1. Effects of GS-Rb1 on Hypoxic- and Ischemic-Induced Damaged Cardiomyocytes

**Figure 1:**
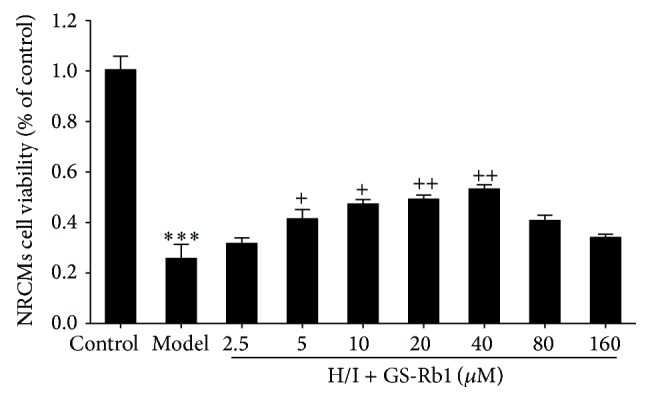
Protective effect of GS-Rb1 on H/I-induced NRCMs death. NRCMs were copretreated with or without GS-Rb1 during H/I condition for 12 h. Cell viability was determined by MTT assay. Error bars represent mean ± SD. Error bars represent mean ± SD. ^***^ *P* < 0.001 versus control, ^+^ *P* < 0.05, and ^++^ *P* < 0.01 versus H/I group (*n* = 3).

Ginsenoside plays important roles in physiological and pathological conditions of various cells. In this research, neonatal rat cardiomyocytes were treated under hypoxia and ischemic conditions in the presence or absence of GS-Rb1. As shown in Figure 1, addition of GS-Rb1 significantly decreased cell death in a dependent manner at an optimal concentration of 40 *μ*M, indicating that GS-Rb1 has a protective role in hypoxic- and ischemic-induced damaged myocardial cells.

### 3.2. Effects of GS-Rb1 on Cell Apoptosis of Cardiomyocytes

**Figure 2:**
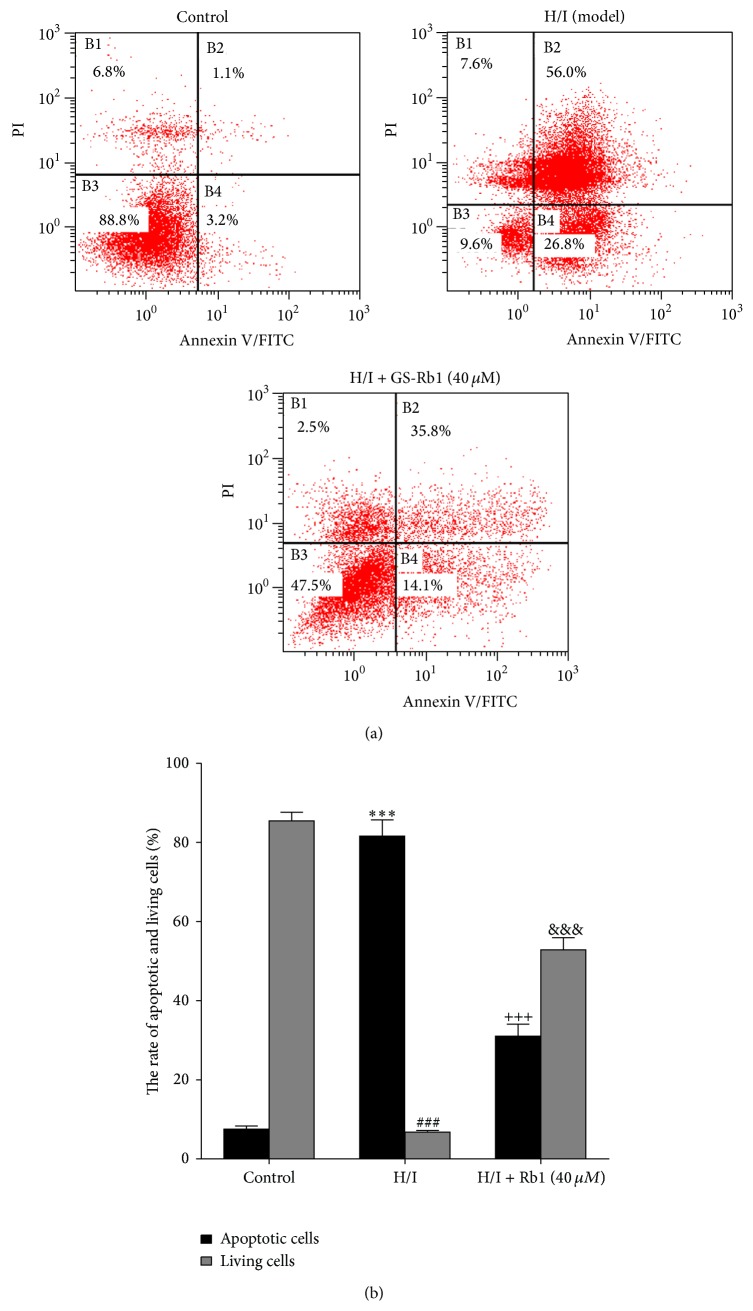
Flow cytometry analysis of GS-Rb1 on cell death induced by H/I. (a) NRCMs were cotreated with or without GS-Rb1 (40 *μ*M) during H/I for 12 h and stained with Annexin V-FITC/PI. (b) Quantification of the percent of apoptotic and living cells in each group. Error bars represent mean ± SD. ^***^ *P* < 0.001 versus apoptotic cells in control group; ^###^ *P* < 0.001 versus living cells in control group; ^+++^ *P* < 0.001 versus apoptotic cells in H/I group; ^&&&^ *P* < 0.001 versus living cells in H/I group (*n* = 3).

To examine the effect of GS-Rb1 on the cell death induced by H/I, Annexin V-FITC /propidium iodide (PI) double-staining assay of cells was analyzed by flow cytometry (Figure 2). The percentage of apoptotic cells (including early and late apoptotic cells) markedly increased in H/I group compared to the control group. With GS-Rb1, apoptosis accounted for 31.0 ± 1.8% at 40 *μ*M GS-Rb1, with the surviving cells increasing 52.8 ± 3.1%. These data indicate that GS-Rb1 can inhibit hypoxic- and ischemic-induced cell apoptosis of neonatal rat cardiomyocytes.

### 3.3. Expression of MicroRNAs in Hypoxic- and Ischemic-Treated Cardiomyocytes

**Figure 3:**
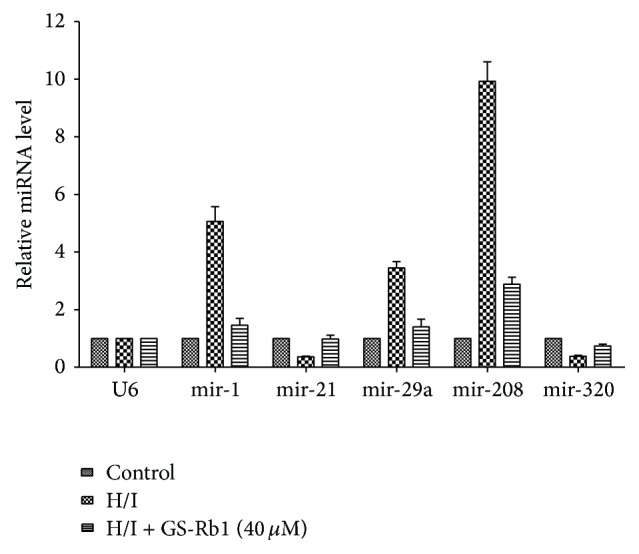
Real-time PCR analysis of GS-Rb1 on miRNAs expression change induced by H/I. NRCMs were cotreated with or without GS-Rb1 (40 *μ*M) during H/I for 12 h. Detection of miRNA expression was performed using poly(A) tailing SYBR Green real-time PCR. The relative expression of miRNA was calculated based on the formula: 2^−(ΔCtmiR−ΔCtU6)^. Error bars represent mean ± SD.

The level of microRNAs was detected by real-time RT-PCR assay. The expression level of mir-1, mir-29a, and mir-208 was increased in the H/I group (5.9-, 3.4-, and 9.3-fold versus control, relatively), while that of mir-21 and mir-320 was significantly decreased (0.35- and 0.41-fold versus control, relatively). With the treatment of GS-Rb1, the expression change of miRNAs in H/I group could be reversed in a certain extent (Figure 3).

## 4. Discussion

GS-Rb1 is one of the most important active compounds of ginseng, and it has multiple pharmacological actions [11–14] and the protective effects of different organs [15–20]. Furthermore, GS-Rb1 has been proved to have cardiovascular protective effect by considerable research.

MicroRNAs (miRNAs) are a kind of conservative single stranded noncoding RNA molecules found in animals, plants, virus, and other organisms and are important regulators of gene expression and fundamentally impact on cardiovascular functions. In recent years, investigations found that miRNAs play an important role in the development of cardiovascular diseases and may become a possible new target in the treatment of cardiovascular diseases. Some researchers have revealed that the miRNAs could be the targets of traditional Chinese medicine to protect cardiovascular system. Tanshinone IIA, a lipid-soluble pharmacologically active compound extracted from the rhizome of traditional Chinese herb* Salvia miltiorrhiza*, has been reported to improve hypoxic cardiac myocytes and postinfarction rat cardiomyocytes by regulating mir-133 and mir-1 and MAPK pathways [21, 22]. In the present research, we have found that GS-Rb1 could protect primary cardiomyocytes from hypoxia and ischemia injuries by reducing cell apoptosis and modulating circulation of miRNAs. Compared with that of the control group, expressions of mir-1, mir-29a, and mir-208 obviously increased in the experimental model groups. In contrast, expressions of mir-21 and mir-320 were significantly downregulated and GS-Rb1 incubation in model group could reverse the differences in a certain extent.

Specifically, mir-1 is skeletal and cardiac muscle specific microRNA necessary for postmitotic muscle proliferation and differentiation [23]. Many researchers have proved that mir-1 plays an important part during cardiac apoptosis [24, 25]. Overexpression of mir-1 could exacerbate cardiac injury; on the contrary, knockdown of mir-1 significantly attenuated cardiac ischemia/reperfusion injury [26]. Mir-29 has shown relationship with cancer cell apoptosis by activating p53 [27]. Downregulation of mir-29 (mir-29a and mir-29c) by antisense inhibitor also protected H9c2 cardiomyocytes from simulated IR injury. Antagomirs against mir-29a or mir-29c significantly reduced myocardial infarct size and apoptosis in hearts subjected to IR injury [28]. Our results demonstrated that GS-Rb1 suppressed the expression of mir-1 and mir-29a in the model group, which might be the microRNA targets of GS-Rb1 to protect cardiomyocytes from H/I injuries.

Mir-21 is another cardiac enriched microRNA, which has been proved to be involved in ischemic heart disease, myocardial remodeling, and vascular proliferative diseases [29]. Overexpression of mir-21 plays an important role during cardiomyocytes apoptosis and ischemia/reperfusion- (I/R-) induced heart damages [30, 31]. Mir-320 has the familiar function with mir-21. Mir-320 expression was significantly decreased in the hearts on ischemia/reperfusion* in vivo *and* in vitro*. Overexpression of mir-320 enhanced cardiomyocyte apoptosis and increased extent of apoptosis and infarction size in the hearts. Conversely,* in vivo* treatment with antagomir-320 reduced infarction size and showed cytoprotective [32]. Our results showed that mir-21 and mir-320 significantly decreased in the H/I injured cardiomyocytes and increased by GS-Rb1 treatment following H/I, which suggested that mir-21 and mir-320 might be the potential microRNA targets of GS-Rb1 to protect cardiomyocytes.

Mir-208 is produced exclusively in the heart. A growing number of studies have demonstrated that mir-208 could be selected as a possible biomarker of myocardial injury and myocardial infarction [33, 34]. Plasma mir-208 increased significantly after isoproterenol-induced myocardial injury and showed a similar time course to the concentration of cTnI [35]. Therapeutic inhibition of mir-208a by systemic delivery of antisense oligonucleotide could improve cardiac function and survival during hypertension-induced heart failure [36]. But there was no enough evidence shown that overexpression or knockdown mir-208 was related to the hypoxia/ischemia injuries and cardiomyocytes apoptosis. Our results showed that GS-Rb1 could suppress the expression of mir-208 in model group. The relationship of mir-208 and cardiomyocytes injury or cell apoptosis needs to be further studied.

In conclusion, GS-Rb1 protects hypoxic- and ischemic-induced damaged myocardial cells by regulating expression of miRNAs, which not only is important in uncovering mechanism of GS-Rb1 in hypoxic- and ischemic-induced damage of myocardial cells, but also may provide a new method for treating myocardial diseases in clinical applications.
